# Conditional monogyny: female quality predicts male faithfulness

**DOI:** 10.1186/1742-9994-9-7

**Published:** 2012-04-25

**Authors:** Klaas W Welke, Stefanie M Zimmer, Jutta M Schneider

**Affiliations:** 1 Zoological Institute, University of Hamburg, Martin-Luther-King-Platz 3, 20146, Hamburg, Germany

**Keywords:** Monogyny, Polyandry, Mate choice, Alternative reproductive tactics, Sexual cannibalism, *Argiope bruennichi*

## Abstract

**Introduction:**

Male monogyny in the absence of paternal investment is arguably one of the most puzzling mating systems. Recent evidence suggests that males of monogynous species adjust their life-history and their mating decision to shifting spatial and temporal selection regimes. In the cannibalistic wasp spider *Argiope bruennichi* males can be either monogynous or mate with a maximum of two females. We studied factors underlying male mating decisions in a natural population over a whole mating season. We documented all matings and categorized the males into single-mated and double-mated monogynous as well as bigynous males.

**Results:**

We found that all categories were continuously present with relatively stable frequencies despite changes in the operational sex ratio. Males were more likely monogynous when copulating with relatively heavy and old females and otherwise bigynous.

**Conclusion:**

Our results imply that males make conditional mating decisions based on the quality of the first female they encounter but do not adjust their mating tactic to the local selection regime.

## Introduction

In males the variety of mating tactics is high and often different tactics are associated with an intra-sexual morphological polymorphism [1,2]. In many species small males use a ‘sneaker’ or ‘parasitic’ tactic to get access to females that are guarded by larger, ‘bourgeois’ males [3,4]. Furthermore, male mating tactics may differ in their optimal mating rate. In species with traditional sex roles, males are known to maximize their fitness by increasing their mating rate whereas multiply mating in females does not necessarily elevate fitness [5,6]. However, in many cases males mate at a lower than maximum rate, for example when they provide parental care [7]. These deviations from traditional sex roles are of particular interest for the understanding of mating system evolution and the expression of alternative tactics. While sex role reversal and bi-parental care is well explained by the general theory [8,9], low male mating rates with low or even no paternal care (here called monogyny) are less well understood. By theory, monogynous mating systems are suggested to evolve if they are associated with a highly efficient paternity protection such that the fertilization success of monogynous males is higher than the average of a polygynous strategy [10]. The evolution of paternity protection, however, only makes sense under a male biased effective sex ratio (ratio between males and females that mate at least once) and a high degree of sperm competition within a species.

Monogyny can be found within several taxa such as insects [11,12] and fishes [13] but is particularly common in spiders, especially web spiders, where it evolved several times independently [14]. Monogyny is associated with curious adaptations like life-long associations between males and females [13], extreme sexual size dimorphism [14,15], genital damage [16-18], and sexual cannibalism [19]. A well known spider example for monogyny is the black widow spider *Latrodectus hasselti.* Males of *L. hasselti* sacrifice themselves to the female by somersaulting into her fangs during copulation [20]. By doing this males are able to increase their copulation duration and thus their paternity share [21]. Monogynous spider species are well known for their peculiar genital morphology and species from the genera *Argiope* and *Nephila* are well studied in this aspect [18,22-25]. Spider males possess two secondary copulatory organs, the pedipalps, which are often damaged during copulation and each pedipalp can only be used once in a lifetime (one shot genitalia) [14,26-29]. In many species these broken-off genital parts serve as mating plugs within the paired insemination ducts of the female and increase male fertilization success under sperm competition as is the case in *Argiope bruennichi*[18]. Hence, due to their genital morphology these spider males are limited to a maximum of two copulations in their lifetime while females can mate multiply. Thus, considering the mating rate, a male has the choice between two main reproductive tactics: (i) they may invest everything into a single female and mate monogynously or (ii) they may copulate with two different females and mate bigynously. While monogynous males are able to completely monopolize a female by plugging both of her genital openings, bigynous males only partly protect their paternity in a single mating because they will always leave one of the spermathecae unused and available for rival males.

In a mathematical model Fromhage et al. [30] have shown that monogyny and bigyny can coexist within populations as alternative tactics of a mixed strategy. Fromhage et al. [30] proposed the two mating tactics of orb-web spiders (monogyny and bigyny) to be conditional reproductive tactics and indeed there is accumulating evidence that males of monogynous spider species are plastic in their behavioral or physiological response to local selection regimes. A recent study on *L. hasselti* demonstrated a remarkable adaptive plasticity in monogynous males as the male maturation could be induced by the presence of female pheromones at a cost of body size and condition [31]. *A. bruennichi* males were shown to alter their mating behavior depending on the presence or absence of female pheromones [32]. Indeed, male spiders detect female mating status through pheromones that virgin females emit [33-35] and the presence of such cues may provide a male with information about local competition and current and future mate availability. Thus pheromones may have an impact on the choice of the best reproductive tactic.

A common measurement for local competition is the operational sex ratio (OSR: ratio of sexually active males to fertilizable females at a given time; [8,36]). The OSR is said to have an influence on the expression of alternative reproductive phenotypes and often underlies variation during and within mating seasons. Especially in monogynous orb-web spiders the OSR changes drastically throughout the mating season. Most of these species are seasonally breeding with one generation per mating season and a first male advantage. As a consequence, protandry (early male maturation) is favored by selection because it increases a male’s chance of encountering virgin females. Here the OSR will initially be male biased and this bias will decrease during the season as mated males disappear from the mating pool due to genital damage and sexual cannibalism, while females continue to mature. Only few studies have yet investigated empirically which factors determine whether a male is monogynous or bigynous [32,37] and no study has done this under field conditions.

Here we investigated under natural conditions which factors influence males of the orb-web spider *A. bruennichi* in their mating decision, namely whether to invest maximally in a single female or whether to opt for inseminating two females. We used a small confined population where it was possible to closely observe all females as well as the roving males during a whole mating season. We inspected all females in this population before the mating season started and removed them from the study site shortly before their final molt. After females matured in the laboratory we brought the freshly molted virgins (maturity reached at most 24 hours before) back to the field and observed all visiting and copulating males and recorded courtship and mating behavior. We determined the frequency of alternative reproductive tactics throughout the mating season and the cues by which males adjust their mating behavior. We predicted varying frequencies of bigynous males as the season and the OSR changes from male biased to female biased as well as between patches with different densities of virgin females. Furthermore, we expected physical condition of males and the value of mating partners to influence males in their mating decision.

## Materials and methods

### Study species

The European wasp spider *Argiope bruennichi* (Scopoli 1772), an entelegyne orb weaver, is present in Europe from the Mediterranean up to Scandinavia. It inhabits dry meadows as well as marsh areas with high densities of crickets as main prey items. *Argiope bruennichi* is characterized by a strong sexual size dimorphism with much smaller males than females. In the laboratory, females are highly cannibalistic and 80% of males are killed during their first copulation [25]. Genital damage is very common in *A. bruennichi* and occurs in 85% of copulations into unused genital openings [18]. The broken-off pieces remain in the female insemination duct in 97% of the cases and are highly effective in their function as mating plugs [18]. By plugging female insemination ducts males secure their paternity share and the fertilization success of future rivals is drastically reduced [18,38]. However, the two separate sperm storage organs of females can only be inseminated in two separate copulations as males can use only one of their pedipalps at a time [18,25,39].

### Study site

The study was conducted in July and August 2009 on a meadow in Hamburg-Moorfleet, Germany (53°30’38”N, 10°6’4”E). The meadow was bordered by a street on two sides, the motorway, and unsuitable habitat on another. We fenced in the observational area of app. 750 m² with barrier tape to prevent passers-by from crossing it. The study site was divided into nine patches of different sizes. By cutting down the vegetation between the patches we created paths for the observers and prevented spiders from building their webs in this area (see [40]). Male mate search seemed unaffected by our paths because we saw males and threads of roving males crossing it.

### Field observations

Before the mating season started we went to the field site weekly to check the female development. As soon as the first adult females appeared we located all female *A. bruennichi* on the meadow by carefully searching for webs. To improve visibility of webs, we used aerosol cans filled with water. Each female web location was marked with a bamboo stick and was visited daily. During these daily checks, we categorized the developmental state of all sub-adult females on the basis of their epigyne swelling. While young sub-adults have no such swelling, the differentiated genital area protrudes below a cutaneous layer in females close to maturation. Thereby we were able to predict the time of their final molt. Females characterized as very close to their final molt (we ensured to leave the sub-adults in the field as long as possible) were removed from the field and were brought to our laboratory at the University of Hamburg. There we housed them in Perspex frames (30 × 30 × 6 cm), sprayed them with water immediately, and fed them with 1 *Calliphora* sp. and approx. 20 *Drosophila* sp. (daily dosage throughout their laboratory stay). As soon as females had molted to adulthood, between 3.56 ± 1.55 days after capture, we measured their adult weight and marked them individually for recognition in the field on their opisthosoma with moisture-proof, non-toxic color dots (Pelikan PLAKA).

Within 24 hours after maturation, females were taken to their collection site inside the Perspex frames where they were fixed with tent pegs on the meadow. We observed females individually for a minimum of four hours after their release. After this focal observation period, all adult females, mated and unmated, were monitored in intervals of 10–15 minutes for the presence of courting, copulating, or cannibalized males. Copulation durations were recorded using stop watches. Depending on the number of females we brought to the field we were between two and six observers. Each one observed one or several females. Observations ended at sunset when visibility was reduced to a minimum and daily observation periods were about 8 hours. If females were not mated within this period they were returned to the laboratory and were brought back to the field on the following days until they were mated. All mated females remained in the field and were scan sampled as mentioned above for the whole mating season. Most of the females stayed within close vicinity to the place where we had released them. All females were easily identifiable due to their individual color marking.

If males were cannibalized after copulation, we rescued them from the female fangs and stored them in plastic vials until they were frozen in the laboratory at −80°C for further measures. Males that were not cannibalized after copulation were marked with waterproof colors (Pelikan PLAKA) on their opisthosoma using a fine brush or a blade of grass as soon as they left the female’s web. Sometimes we painted a male’s leg or parts of his prosoma but they continued their mate search unaffected. Additionally, we noted for each male its number of legs before and after copulation for individual recognition.

### Mating season

On July 10^th^ we found two freshly molted adult females in the field, one of them copulating, and considered this as the start of the mating season. We took the individuals into the laboratory to remove them from the study site and to avoid further unobserved copulations. From this day on, we started daily trips to the field site. The last sub-adult male in the field was sighted on the 24^th^ of July 2009 and the last copulation was seen on the 7^th^ August. Hence, the mating season ended after 29 days. The last adult female (adult: 07.08.2009) did not mate with any male. Overall we observed 98 females, although 121 females were on our study site, and collected 128 males (one male was lost).

### Laboratory work

In the laboratory, we measured tibia-patella-length of the first pair of legs and prosoma length of all collected males under a dissecting microscope using the measuring tool of Leica IM500. Additionally, we examined each male’s pedipalps for possible breakage damage. Because we knew from our observations which pedipalp a male had used for copulation we were able to combine the data of color marking, number of legs, and used pedipalp to identify individual males when they approached a new female. We used these data to assign males to either the monogynous or the bigynous mating tactic. Males could be monogynous because (i) they were killed and cannibalized after their first copulation (forced monogyny **M1**) or because (ii) they mated twice with the same female (voluntarily monogynous **M2**). Bigynous males mated two times with different females (**B**). Males were classified as bigynous whenever they had clearly survived their first copulation and continued mate search, even if we did not witness one of his two copulations (see results for details). For example, among observed copulations with the result of sexual cannibalism, several males had both of their pedipalps damaged clearly indicating an unobserved previous copulation with a different female. If data for pedipalp use were missing we excluded this individual from the analyses of mating tactic.

For calculating the OSR we divided the number of adult females (mated and virgin) in the field on each day by the number of adult males on or close to female webs plus those males that had courted or mated with females during that day.

Besides the OSR we also measured the distance of each female to her closest female neighbor and we determined the total number of females within a radius of 1, 2, 3, and 5 meters.

The reproductive success of each male was estimated by calculating the paternity share of each male with his female(s). The paternity share of males that had mated with polyandrous females can be determined with high accuracy from the copulation duration of each male. The relationship between copulation duration, the use of plugged or unplugged copulatory ducts and sperm transfer as well as relative paternity are well known for *A. bruennichi*[18,25,38]. A male’s reproductive success depends on his duration of copulation and that of his rival(s). Therefore we translated the copulation durations of rival males into percentages and used this to calculate paternity shares. Furthermore paternity is influenced by the presence of mating plugs. Past studies have shown that the presence of a mating plug reduces the paternity share of a successor to approx. 10% [18,38]. Therefore we allocated males a paternity share of 10% if they had copulated into a plugged genital opening. By taking all this into account we were able to estimate individual paternity shares.

### Statistical analyses

Statistical analyses were conducted using the JMP IN 7.0.2 for Windows (SAS Institute Inc., Cary, NC, USA). Data were tested for the distribution of the residuals and parametric statistics were applied if the requirements were fulfilled. Otherwise we used a t-test for unequal variances, a Welch-Anova, a Kruskal-Wallis Anova (*X*²), Mann–Whitney-U test (*z*), or Chi-square test (*X²*_*2*_) as implemented in JMP. All tests are two-tailed. All data are presented as mean ± standard deviation.

## Results

### Male mating rates

**Figure 1:**
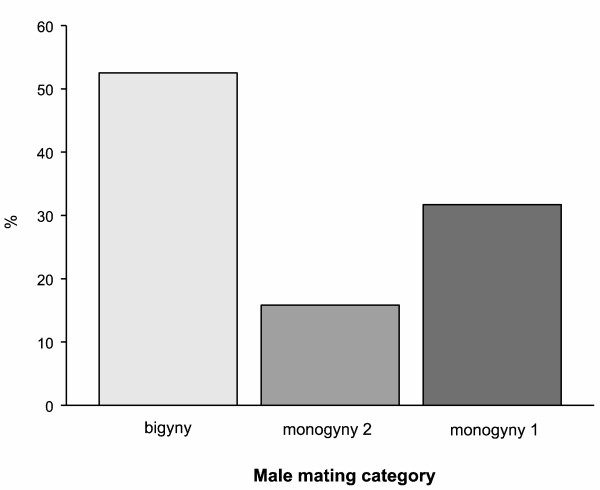
Frequencies of male mating tactics (monogyny 1 copulation, monogyny 2 copulations, bigyny) in the focal population.

The ratio of monogynous to bigynous males was balanced in our study population with 52.5% that mated with two females (bigynous B) and 47.5% with one (monogynous M) (*N* = 101, Figure 1).

We observed bigynous males either during their first (*N* = 27, 4 males fled before marking) or their second copulation (*N* = 22) but rarely during both: 18.5% (5 out of 27) of color marked bigynous males were recaptured after their first copulation and were unambiguously assigned to both of their mating partners. On average these males had covered a distance of 6.5 ± 5.4 m (range 4-16 m) between the two females and the journey took them 2.4 ± 2.7 days (range 0–7 days).

The monogynous males either copulated once (M1, males cannibalized after a single copulation; 32 of 48) or twice (M2: 16 of 48) with the same female. In the M1 group it cannot be decided whether males would have followed a bigynous or a monogynous tactic if only they had survived their first copulation. Hence, for analyses we divide monogynous males into those that were voluntarily monogynous with two copulations (M2) and those whose behavioral decision was unclear because they were cannibalized after a single copulation (M1).

### Male features

A male may base his decision whether to continue mate search or to re-mate with the same female on estimations about his own phenotype and physical condition. However, no measurement of male size (tibia-patella length, prosoma length) predicted the mating tactic (Table 1). Which pedipalp he used in the first copulation and whether he damaged his genitalia or not was also not relevant for his mating decision (Table 1).

**Table 1:** Male phenotypes, mating behavior, and female features of the three male reproductive tactics

	**Monogyny** **(1 copulation)**	**Monogyny** **(2 copulations)**	**Bigyny**	**Test statistic**	***P***
**Duration of 1**^**st**^**cop (s)**	8.9 ± 4.21 (25)	6.61 ± 2.62 (14)	7.14 ± 2.82 (32)	Welch-Anova: *F*_2,68_ = 2.3	0.12
**Duration of 2**^**nd**^**cop (s)**	-	68.78 ± 34.14 (15)	62.47 ± 78.62 (22)	*X*² = 2.69	0.1
**Pedipalp used in 1**^**st**^**cop**	59.4% right (19 of 32)	40% right (6 of 15)	46.8% right (22 of 47)	*X*²_2_ = 1.93	0.38
**Genital damage 1**^**st**^**cop**	Big:12.5%	Big:20%	Big: 13%	*X*²_2_ = 4.41	0.35
	Small:87.5%	Small: 73.3%	Small:78.3%		
	None: 0%	None:6.7%	None: 8.7%		
	(32)	(15)	(23)		
**Genital damage 2**^**nd**^**cop**	-	Big:13.3%	Big:8.7%	*X*²_2_ = 2.6	0.27
		Small:80%	Small:65.2%		
		None:6.7%	None: 26.1%		
		(15)	(23)		
**♂♂ tibia-patella-length (mm)**	4.0 ± 0.45 (20)	3.95 ± 0.36 (14)	4.1 ± 0.56 (18)	*F*_2,49_ = 0.39	0.68
**♂♂ prosoma length (mm)**	2.4 ± 0.2 (31)	2.4 ± 0.13 (16)	2.45 ± 0.25 (24)	Welch-Anova: *F*_2,68_ = 0.77	0.47
**♂♂ no. of legs before 1**^**st**^**cop**	7.13 ± 1.06 (23)	7.23 ± 1.17 (13)	7.29 ± 0.98 (28)	*X*² = 0.29	0.87
**1**^**st**^**♀♀ adult weight (mg)**	149.9 ± 65.7 (32)	130.1 ± 48.8 (16)	103.8 ± 40.1 (34)	*X*² = 9.43	**0.009**
**2**^**nd**^**♀♀ adult weight (mg)**	-	128.9 ± 50.27 (15)	133.3 ± 50.6 (24)	*z* = −−0.07	0.94
**1**^**st**^**♀♀ age at cop (days)**	2.8 ± 2.6 (32)	1.4 ± 1.2 (16)	1.0 ± 0.8 (34)	*X*² = 20.28	**<.0001**
**2**^**nd**^**♀♀ weight at cop (mg)**	-	1.4 ± 1.2 (15)	2.1 ± 2.8 (24)	*z* = 1.16	0.25
**1**^**st**^**♀♀ mating status**	21.9% mated (7 of 32)	6.3% mated (1 of 16)	17.7% mated (6 of 34)	*X*² = 2.17	0.34

Sample sizes are given in parentheses. Mean ± standard deviation is shown for all data. *F =* ANOVA*, X² =* Kruskal-Wallis-test, *z* = Mann–Whitney-U test, *X*²_2_ = Chi-square test. Significant differences (*P* < 0.05) are given in bold.

Interestingly, males with legs missing at their first copulation were found more frequently early in the season than later (linear regression: *r²* = 0.12, *F*_1,74_ = 10.54, *P* = 0.002). The decline in injuries with progressing season coincides with a decline in male competition inferred from the changes in the OSR. Indeed, there is a significant negative relationship between the male bias in the OSR and the number of legs males lost at their first copulation (*r²* = 0.12, *F*_1,74_ = 9.9, *P* = 0.002).

The duration of a male’s first copulation is directly and positively related to the probability of sexual cannibalism (*t*_71_ = 2.06, *P* = 0.047) and thereby influences a male’s future mating opportunities. However, the differences in copulation durations are not significant if we compare the three mating categories (Table 1). Only within the monogynous males, we found that M1 males copulated slightly longer than M2 males (*t*_39_ = −2.09, *P* = 0.04). Overall we observed a rather low frequency of cannibalism in the first copulation of 31.4% (32 of 102) if compared to previous studies [25,41].

### Female features

Most first copulations of males (90%) occurred with virgin females (*X²*_*2*_ = 38.7, *N* = 82, *P* < .0001) and this preference was found in all three mating categories (*X²*_*2*_ = 2.17, *N* = 82, *P* = 0.34). Interestingly, first copulations of males with mated females were least frequent in the M2 category (Table 1). The frequency of copulations with mated females of bigynous males increased non-significantly from 17.7% in first matings to 29% in the second matings (7 of 24, *X*²_2_ = 0.67, *P* = 0.41).

A mated female will have at least one genital opening plugged and a male that copulates with a mated female risks to copulate into an already plugged genital opening and to gain no paternity share. In 10 out of 14 cases in which males mated with non-virgin females we were able to determine whether they copulated into a used or a virgin genital opening. Three of those males mated with a double-mated female and therefore had no chance to insert their pedipalp into a virgin genital opening. The remaining 7 males mated with single-mated females and only 4 of them successfully avoided the used genital opening. This finding corroborates earlier observations that males are not able to determine the unused genital opening in a half-sided virgin [18].

**Figure 2:**
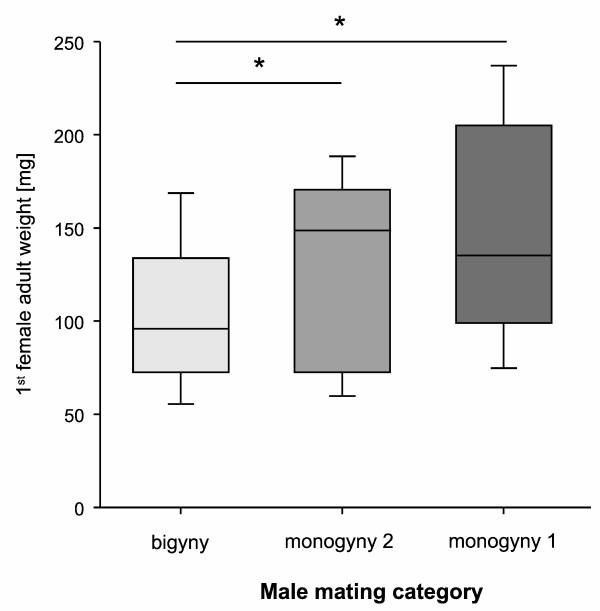
**Female adult weight of first mating partners of males.** Males were more likely to be monogynous when mating with a heavy female first.

**Figure 3:**
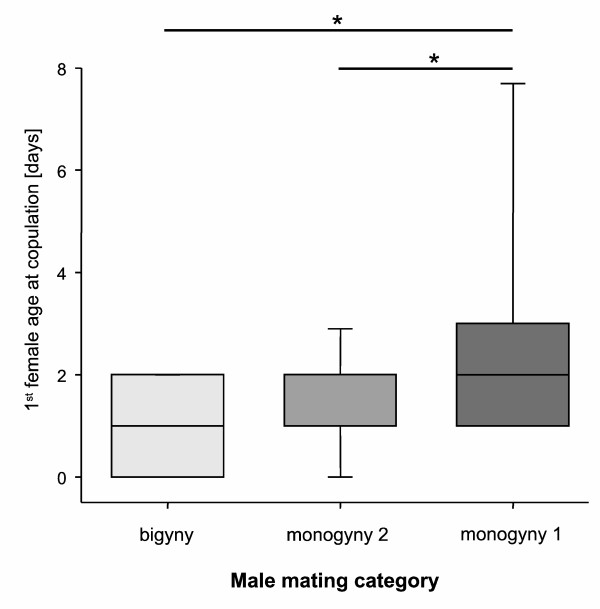
**Female adult age of first mating partners of males.** Single-mated monogynous males mated with the oldest females.

The weight of a female had significant influence on the male mating categories (Table 1; Figure 2) and post-hoc pairwise comparisons of the categories revealed significant differences between bigynous males and both monogynous categories (M1 and B: *z* = 3.03, *N* = 66, *P* = 0.003; M2 and B: *t*_50_ = 2.02, *P* = 0.049; Figure 2). Furthermore, 2^nd^ mates of bigynous males were significantly heavier than 1^st^ mates (*t*_58_ = 2.48, *P* = 0.016) but did not differ in age (*z* = 1.16, *N* = 58, *P* = 0.25). But the age of a female influenced the likelihood of sexual cannibalism: females that had cannibalized single-mated M1 males were significantly older than females of males that survived (M2, B; Table 1, Figure 3). First females of M2 and B males were not different in age (M2 and B: *z* = 0.87, *N* = 50, *P* = 0.39).

### Temporal and spatial selection regimes

**Figure 4:**
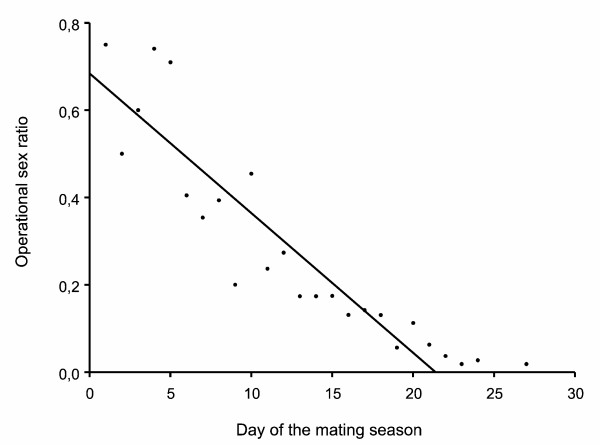
The operational sex ratio given as the ratio of adult males and adult females regardless of their mating status during the course of the season.

The time of season had no significant influence on male mating tactics (date of a male’s first copulation: ANOVA: *F*_2,79_ = 1.19, *P* = 0.31; adult date of the first female: *X*² = 2.63, *n* = 82, *P* = 0.27). Although we found a change in the operational sex ratio during the course of the mating season (Figure 4; *r²* = 0.82, *F*_*1,23*_ = 104.4, *P* < 0.0001) this had no influence on male mating tactics (nominal-logistic regression: *r²* = 0.01, *X²* = 1.86, *N* = 82, *P* = 0.39). Only time of day had an effect. The first copulation of M2 males happened at an earlier time of day than those of M1 and B males (*X*² = 6.17, *N* = 81, *P* = 0.05). This difference is more pronounced when comparing the M2 and the B males only (*z* = −2.44, *N* = 49, *P* = 0.01).

Also the spatial distribution of females had no influence on male mating decisions. A multivariate nominal logistic model revealed that measurements of female availability in a 3 meter radius around the 1^st^ mating partner of a male, explained none of the variation in male mating tactics (nominal-logistic regression: *r*² = 0.04, *X*² = 7.35, *P* = 0.29; individual factors: number of sub-adult females: *X*² = 3.22, *P* = 0.2, number of virgin females: *X*² = 2.21, *P* = 0.33; number of mated females: *X*² = 1.75, *P* = 0.42). Additionally, the number of potential mating partners (irrespective of their developmental status) surrounding the 1^st^ female of a male had no influence on its mating category (2 m radius: *X*² = 0.17, *N* = 83, *P* = 0.92; 5 m radius: *X*² = 3.58, *N* = 83, *P* = 0.17); neither did the distance of the 1^st^ mate to its nearest female neighbor (*X*² = 1.14, *N* = 80, *P* = 0.57). Interestingly, the developmental status of the nearest neighbor differed between categories (*X*²_2_ = 12.27, *N* = 67, *P* = 0.02) and revealed that the 1^st^ mate of B males was mostly neighbored by mated females (75%) while 60% of the 1^st^ females of M2 males were neighbored by sub-adult females (*X*²_2_ = 4.36, *N* = 39, *P* = 0.04).

### Reproductive success

M1 males monopolize only one copulatory opening of a female but leave the other one available for rivals. Only 3 of 22 M1 males shared a female with a successor and thereby lost between 10-90% of the paternity. The remaining 19 M1 males secured 100% paternity. The average paternity of M1 males was 90.8 ± 25.3%.

M2 males secure 100% paternity if they mate with a virgin. But their paternity share is reduced if they mate with an already mated female which occurred in one case, or if a successor copulated with their female which happened in 5 out of the 15 cases. The average paternity of M2 males was 90.7 ± 20.5%.

B males ideally monopolize both of their females but may also lose paternity shares with both of them. Estimating reproductive success in this category is difficult because of missing data for either the first or the second mating partner of a male. The combined data of those males that provided us with paternity shares of either their first or second female average paternity with the first female of 65.1 ± 42.4% (*N* = 8) and with the second females 53.2 ± 42.7% (*N* = 8) in sum they fertilized the equivalent of 118.3% of a single female’s eggs.

Only 4 of the B males provided us with data for both mating partners and their paternity share ranged from 20%, when the male invested both his copulations into already plugged genital openings, up to a maximum of 200% where both females were virgin and did not re-mate. The average paternity of those 4 bigynous males was equivalent to 150.1 ± 87% of a single female’s eggs. Hence, we have good indication that the bigynous mating tactic has the highest variance in paternity share.

## Discussion

We determined the frequency of monogynous and bigynous males in a natural population of *A. bruennichi* and those factors that influenced males in their choice for the best mating tactic. Monogyny and bigyny occurred in almost equal proportions in the study population and we found no temporal pattern. Hence, the hypothesis that males adapt their mating tactic to the changes of local competition was not supported. In contrast, males made state-dependent decisions based on female mating status, age, adult weight, local availability of further mating partners, and the time of day. Males were more likely to mate twice with the same female (monogyny) if it was early in the day, if the female was heavy and the next neighboring female was sub-adult. Males kept on searching for a second female (bigyny) if it was late in the day, the first female was light and the neighboring female was already mated. We found that bigynous males traded up to heavier females as second mates but showed an increased tendency to copulate with already mated females in these copulations. Interestingly, males that died after their first copulation had copulated with heavy females similar to the monogynists that copulated twice although mates of the former were older. These findings imply that male mating tactics are the result of behavioral plasticity and state-dependent decisions rather than being alternative reproductive tactics of a genetically fixed strategy.

Surprisingly we found both monogynous and bigynous males in similar frequencies throughout the season and detected no shift with the hypothesized changes in levels of male-male-competition. Due to protandry, the operational sex ratio (OSR) changes over the course of the season as males are removed from the mating pool via sexual cannibalism. Early in the season most males compete for the first maturing females while this can shift to the opposite pattern late in the season. Therefore we expected frequency shifts in male mating tactics over the course of the mating season.

The only temporal effect on male mating tactic we found was that first copulations of double-mated monogynists happened earlier during the day than those of bigynists. The time of the day could have an influence on a male mating decision because males that find a mating partner during the end of the day may delay their second copulation to one of the following days. This can only be true if males of *A. bruennichi* avoid matings during the night time which is in accordance with recent 24-hour field observations in which all but one copulation was observed during the day (SMZ, personal communication).

Body size can have a large impact on a male's reproductive tactic and generally influences his ability to compete for mating partners. Often the expression of different mating tactics such as sneakers and territorial males are determined by male size [1,2]. In *A. bruennichi* we found no size differences between monogynous and bigynous males. Interestingly the number of legs a male had left was directly related to changes in the OSR during the mating season. While in the beginning of the season with a male biased OSR competition among males was high and males were frequently found with less than eight legs, this changed towards the end of the season when the OSR became female biased. Here males were more likely found with all their legs left. This supports theory that the OSR is a good indicator of intra-sexual competition within a population [8] and that males in our study lost their legs during fights with rivals. However, the number of legs a male had left did not influence his choice of mating tactic.

Males in our study were mainly influenced by female quality and showed a clear preference for virgin over mated females. This preference is highly adaptive especially in a mating system with effective mating plugs and limited male mating opportunities [42-45] and has also been shown for males of the congener *A. keyserlingi*[43] as well as for several other species [46-50]. A recent field study on *A. bruennichi*[45] revealed that females lose their attractiveness for males after mating once. Given the high risk of gaining no or low fertilization success with a mated and possibly plugged female this selectivity seems adaptive. The presence of a mating plug decreases a male’s copulation duration [18] and severely limits paternity [38]. Therefore the value of a virgin female is generally high because it enables males to avoid sperm competition and to monopolize a female by plugging both genital openings. Usually the roving males sense the presence of virgin females via airborne and web-bound pheromones [33,51] but due to protandry adult males also secure their access to virgin females by lingering around the webs of sub-adults waiting for their final moult [40]. Some males even court sub-adults, risking to get cannibalized prior to copulation (as observed in one case in the present study). Thus males seem to be eager to approach freshly molted females that are still immobile and defenseless while their exoskeleton hardens. Those opportunistic or “soft-matings” enable males to monopolize a female without the risk of sexual cannibalism. In the congener *A. aurantia* the majority of matings occur while the female is molting [52] but for *A. bruennichi* the natural frequency is yet unknown but investigations are in progress. In our study we excluded this option because females molted in the laboratory, but we observed one soft-mating in the field and saw a male hanging dead but not cannibalized or wrapped in silk next to the female with both of his pedipalps mutilated (KWW, personal observation). Despite the advantages of mating with virgins, copulations with mated females occurred in our study although at moderate frequencies. We had expected a high variance in the degree of polyandry in *A. bruennichi* due to protandry and high competition for females in the beginning of the season, however, Zimmer et al. [40] found that females mated on average with 1.3 different males and all but one female had at least one mating partner.

Besides female mating status, males were mainly influenced by female weight in their choice for the best mating tactic. Weight positively predicts female fecundity and thus consequently impacts on male reproductive success. Similar male mate choice preferences have been found in several insect and spider species [53-56]. In our study, males that mated with relatively heavy females first were more likely to follow a monogynous than a bigynous mating tactic. Bigynous males may compensate this disadvantage by selecting a relatively heavy female as their second mating partner and may follow a tactic of first securing a successful insemination regardless of female quality before they move on to search for a high quality second partner. This interpretation may explain results from an earlier field study on *A. bruennichi* in which a small proportion of males inspected two given females and mated with the heavier one, while the majority of males mated with the first female they encountered regardless of whether a heavier female sat right next to her [45]. In a natural setting, virgin females of *A. bruennichi* are regularly rejected by some males [40] while others stay to mate; an observation that is difficult to explain given that males compete for access to virgins (see above) [42]. These counterintuitive male mating decisions may be explained by the coexistence of alternative mating tactics: one tactic is to mate with the first available female while the alternative is to accept only females with particular characteristics. Alternatively or additionally, at least a proportion of the selective males observed in the field may have been already mated bigynists in search for a second mating partner (male mating status was not assessed in [45]).

Some of the monogynous males were cannibalized after their first copulation while others used both of their mating options with the same female. While the latter can be interpreted as a clear-cut male decision, interpreting the decisions of single-mated males is more difficult. At least some of these males may have been forced into monogyny by aggressive females while others may have chosen to sacrifice themselves to their mating partner already during their first copulation. Whether a male falls victim to sexual cannibalism during the first copulation is a direct function of its duration; any copulation longer than 10 seconds will most likely end with the death of the males while males that jump off before 10 seconds have a relatively high chance of surviving [25]. Indeed, copulation durations of single-mated males show a high variance which suggests that some of them chose to copulate for longer in spite of sexual cannibalism being the likely consequence, while others attempted to escape but failed. Single-mated monogynists mated with the heaviest and oldest females of all male behavioral categories. High adult weight indicates high fecundity, and adult age indicates that oviposition will occur soon. Both traits benefit male reproductive success and may hence favor a monogynous strategy with a safe option of monopolizing a single spermatheca only but with the maximal sperm transfer [32,57]. Alternatively, older females may be more aggressive and more successful in capturing and cannibalizing a male that attempts to escape.

The high frequency of single-mated males increased the success of bigynous males in the study population. Bigyny would not have evolved if all males copulated twice with a female especially in a species with such a highly effective paternity protecting mechanism. Males in *A. bruennichi* place their plug in about 85% of the copulations into unused genital openings and thus reduce the risk of direct sperm competition [18,32]. However, single-mated females (as well as virgin females) are still available for insemination, offering sufficient mating opportunities on which bigynous males can capitalize. A bigynous mating strategy should only be viable if a large enough proportion of females mate only once supporting the theoretical prediction of frequency dependent co-occurrence of monogynous and bigynous mating strategies: with increasing numbers of bigynists, more females will receive multiple copulations so that mean reproductive success as well as its variance will drop [30].

Based on a male's copulation duration and genital damage we were able to calculate the paternity share of many observed males. This calculation revealed that both monogynous tactics, single and double mated, gained equal paternity shares (about 90%) while the bigynous males had a higher overall paternity share with their females (about 130%). But the variance in paternity share for bigynous males was higher than for monogynous ones and they risked copulating with two already mated females because they showed an increased affection to mated females. Thus bigynous males opt for the mating tactic with the highest paternity share but also with the highest variance. These results imply that males use a conditional strategy with three different alternative tactics. Conditional, or state-dependent, reproductive strategies are defined by different reproductive success of their alternative tactics [58]. This seems to be the case in *A. bruennichi* where bigynous males gained on average a higher paternity share than monogynous ones. Theoretical models on the evolution of monogyny in spiders have shown that the alternative reproductive tactics (monogyny and bigyny) can coexist under certain conditions [30]. Fromhage et al. [30] proposed that the two strategies may not be genetically determined but conditional reproductive tactics enabling spider males a plastic response to local selection regimes. According to our results we suggest that the occurrence of mono- and bigyny is due to conditional male mating decisions.

## Conclusions

In conclusion, our study shows that mono- and bigynous mating tactics coexist in a population of spiders with one-shot genitalia. Males appear to make individual decisions based on the value of the first female they happen to encounter and environmental factors such as the mating status of the next available female and the time of day of the first copulation. Temporal fluctuation of operational sex ratios as well as larger scale spatial factors such as female availability within the population did not play a role. Predictions derived from demography alone are likely not sufficient to explain mating tactics. Thus our results imply that males are plastic in their mating decision and can choose simultaneously between different mating tactics. The mating tactics differ in their fitness and are state-dependent suggesting that they are part of a conditional strategy.

## Abbreviations

B, Bigynous males; M1, Single-mated monogynous males; M2, Double-mated monogynous males; OSR, Operational sex ratio.

## Competing interests

The authors declare that they have no competing interests.

## Authors’ contribution

KWW and SMZ carried out the field study. KWW interpreted the results and prepared the manuscript. SMZ and JMS helped interpreting the results and drafting the manuscript. All authors read and approved the final manuscript.
